# Conductor gestures influence evaluations of ensemble performance

**DOI:** 10.3389/fpsyg.2014.00806

**Published:** 2014-07-24

**Authors:** Steven J. Morrison, Harry E. Price, Eric M. Smedley, Cory D. Meals

**Affiliations:** ^1^Laboratory for Music Cognition, Culture and Learning, School of Music, University of WashingtonSeattle, WA, USA; ^2^School of Music, Kennesaw State UniversityKennesaw, GA, USA; ^3^Jacobs School of Music, Indiana UniversityBloomington, IN, USA

**Keywords:** conducting, audio-visual interaction, expressivity, music performance, music ensembles, performance evaluation

## Abstract

Previous research has found that listener evaluations of ensemble performances vary depending on the expressivity of the conductor’s gestures, even when performances are otherwise identical. It was the purpose of the present study to test whether this effect of visual information was evident in the evaluation of specific aspects of ensemble performance: articulation and dynamics. We constructed a set of 32 music performances that combined auditory and visual information and were designed to feature a high degree of contrast along one of two target characteristics: articulation and dynamics. We paired each of four music excerpts recorded by a chamber ensemble in both a high- and low-contrast condition with video of four conductors demonstrating high- and low-contrast gesture specifically appropriate to either articulation or dynamics. Using one of two equivalent test forms, college music majors and non-majors (*N* = 285) viewed sixteen 30 s performances and evaluated the quality of the ensemble’s articulation, dynamics, technique, and tempo along with overall expressivity. Results showed significantly higher evaluations for performances featuring high rather than low conducting expressivity regardless of the ensemble’s performance quality. Evaluations for both articulation and dynamics were strongly and positively correlated with evaluations of overall ensemble expressivity.

## CONDUCTOR GESTURES INFLUENCE EVALUATIONS OF ENSEMBLE PERFORMANCE

Visual information has been shown to play an important role in the perception and evaluation of musical intentionality and expressivity in performance. Physical gesture as a marker in musical practice is strongly linked to all aspects of the performance experience, to the point of movement and music-making being seen as inseparable in several music cultures (Emberly and Davidson, 2011). Indeed, it might be that separating these two aspects of performance creates a different perception of the musical experience being portrayed, such that “a genre is never independent of technologies or mediation processes” (Thompson et al., 2005, p. 206). Although it has been suggested otherwise (McPherson and Thompson, 1998), it appears appropriate to state that, with the possible exception of audio-only recordings, visual information sits as an integral part of musical performance.

Previous research has shown that this visual channel, manifested in physical gesture, evidences that the “muscular reactions that arise while playing music are also carriers of musical expression” (Gellrich, 1988; translated by Parncutt, 1991, p. 177). This has been found in both expressive gestural information communicated by performers (Davidson, 1993; Vines et al., 2006; Dahl and Friberg, 2007; Broughton and Stevens, 2009) and in an audience’s multi-modal perception of the broad assortment of musical information to be observed. This phenomenon encompasses multiple levels of musical sophistication (Schutz, 2008), ranging from broad expressive intentions of the performer (Davidson, 1993; Broughton and Stevens, 2009) to more fine-grained information such as timbral differences between plucked and bowed string instruments (Saldaña and Rosenblum, 1993). Especially illustrative of this visual influence, Schutz and Lipscomb (2007) found that while longer and shorter performance gestures do not affect acoustic measures of percussion note durations, longer gestures resulted in the perception of longer sounding tones due to combined visual and auditory sensory integration. The present study focuses on how the combined visual perception of specific arrays of conductor gestures and aural musical expressivity affect an audience’s perception and evaluation of music performances. Previous research in cross-modal sensory interactions in music has found that motion is embodied in the constructs of tempo and rhythm, although it is also often ascribed to melody and harmony (Shove and Repp, 1995). Indeed, this helps to clarify that the visual stimuli encoded by performers communicate an abundance of expressive information to the listener (Davidson, 1993; Thompson et al., 2005; Vines et al., 2006; Juchniewicz, 2008).

In the tradition of Western Art Music, conductors serve as both physical and conceptual focal points. Within the context of large ensembles, “musical performance is thought of as a one-way system of communication, running from composer to individual listener through the medium of the performer and further mediated by the expressive motions of the conductor” (Small, 1998, p. 6). The perceived quality of gestural communication exhibited by conductors is often the measure by which their skills are evaluated with movement emphasizing beat induction over expressing seen as evidencing a lower level of musical prowess (Leinsdorf, 1982). Beyond the simple maintenance of pulse (beat pattern) and indications of performers’ entrances (cues), conductors utilize more or less horizontal and vertical space, crisp or fluid hand and arm movements, simulation of weight or resistance, and – most critically – variability in the deployment (Byo and Austin, 1994) of these gestures to convey their interpretation of music’s expressive content. The relationships of conductors’ actions (e.g., expressive gesture, frequent and sustained eye contact, varied facial expression) to resultant performances are now beginning to be established (Yarbrough, 1975; VanWeelden, 2002; Johnson et al., 2003; Labuta, 2010; Napoles, 2013). Indeed, certain gestures or *emblems* have been shown to be capable of transmitting specific musical ideas (Sousa, 1989; Byo, 1990; Sidoti, 1990; Mayne, 1993). Other research has found that experienced conductors use more idiosyncratic expressive gestures (Byo and Austin, 1994; Goolsby, 1999). Napoles (2013) compared choral performances under strict and expressive conducting in conditions that included front (ensemble) and rear (audience) perspectives. Regardless of viewing angle, she observed significant differences in evaluations of tone quality, expressivity, and overall impression between strict and expressive conductors. Expressive conducting has also been reported to engender positive attitudes toward performances (Laib, 1993; Sheldon, 2000; Wöllner, 2008; Silvey, 2013), even when there was no change in the performances (e.g., Price and Winter, 1991; Fredrickson et al., 1998; Schutz and Lipscomb, 2007).

As music is a multivariate entity – encompassing a wide range of elements in its performance and construction – traditional conducting pedagogy has largely codified representational gestural vocabularies with which to communicate wide swaths of this information in a one-to-many setting (conductor-to-ensemble). Bender and Hancock (2010) reported a study in which high- and low-intensity gestural conducting was combined with high- and low-performance quality to examine the relationship of these to the evaluation of conductor effectiveness. Generally, the high-intensity conductor was rated higher, but the quality of performance also had an effect on assessment with a high-quality performance resulting in a higher conductor rating. Silvey (2011) reported similar results regarding the effect of ensemble performance quality on evaluations of identical conducting videos.

Recent research has reported the dominance of visual information in evaluations or identifications of musical performance (Tsay, 2013; Mitchell and MacDonald, 2014). Conducting gesture ostensibly seeks to organize and synchronize ensemble efforts to realize a musical performance combining a composer’s expressed intention and the conductor’s internal conception of a given work. The synchronization value embedded in a conductor’s efforts has been examined (e.g., Luck and Sloboda, 2009; D’Ausilio et al., 2012). The degree of expressivity in a conductor’s gestural content has been shown to have a positive correlation with audience perception of general performance expressivity (Morrison et al., 2009; Price and Mann, 2011; Morrison and Selvey, in press; Price et al., in press). Even when ensemble performances did not vary, both musically experienced and inexperienced viewers rated ensemble expressivity to be higher in conditions featuring expressive conductors. While gesture’s general influence on expressivity has become increasingly well documented, its interaction with specific and refined aspects of music performance is, as yet, less well understood.

Within expressivity, articulation and dynamics are notable due to the clarity of their communication in both gestural and notation-based contexts. While conducting itself is not a sound-producing endeavor, it nonetheless benefits from gestural correlates in other areas of musical activity. In drumming, an increase in implement height is seen immediately prior to the playing of a single accented stroke or throughout a louder succession (Dahl, 2000). Similarly, but not explicitly linked to dynamic contrast, increases in motion, especially upper body, were observed to be of high salience to evaluations of expressivity in piano performance (Davidson, 1994). Considered alongside the influence of short- and long-duration gestures on perceptions of acoustic duration (Schutz and Lipscomb, 2007), it becomes clear that the curated gestural vocabulary put forward by Elizabeth Green (Green and Gibson, 2004), though anecdotal in nature, has been corroborated by other phenomena in musical performance. Recent research has begun to clarify conducting gesture’s general influence on expressivity, but its interaction with specific and refined aspects of music performance and perception is, as yet, less well understood.

Here, we examined whether greater specificity in musical/gestural relationships might yield a richer understanding of external perceptions and evaluations of musical experiences. The isolation and manipulation of musical elements that evince a high level of sonic contrast from the ensemble and visual contrast from the conductor – such as articulation and dynamics – may provide a deeper and more textured view of gesture’s interaction with musical performance. In other words, would effects observed at the broad level of expressivity persist in evaluations of two of its more specific constituent parts?

## MATERIALS AND METHODS

We selected four music excerpts; two featuring high levels of dynamic contrast (e.g., piano/forte) and two featuring pronounced contrast of articulation (e.g., legato/staccato). These two musical parameters are uniquely specified in music notation and are associated with a specific and broadly understood vocabulary of conducting gestures (Green and Gibson, 2004; Jordan et al., 2011). Excerpts were extracted from four classical string quartets and rescored for a small chamber wind ensemble (**Table 1**). The 11-member chamber group was selected to create a full ensemble timbre but allow for a high degree of precision in the realization of each excerpt with clear executions of target parameters (articulation, dynamics). Consistent with previous research in this area (e.g., Price and Chang, 2005; Price, 2006), excerpts were of approximately 30 s durations that started and ended at appropriate phrase points, and included an equal balance of dynamic or articulation contrast. Within like pairings, we included one faster and one slower (~75% of faster counterparts) excerpt.

**Table 1:** Performance excerpts.

*Dynamics*

String Quartet no. 17 in Bb Major, K. 458, Mvt. II: Minuetto and Trio (mm. 29-48), Wolfgang Amadeus Mozart
String Quartet Op. 18, no. 1, Mvt. I: Allegro con brio (mm. 1-20), Ludvig van Beethoven

*Articulation*

String Quartet Op. 59, no. 7 ”Rasumovsky”, Mvt. II: Allegretto vivace e sempre scherzando (mm. 1-30), Ludvig van Beethoven
String Quartet No. 2 in Eb Major, Op. 1, no. 2, Mvt. III: Menuetto and Trio (mm. 27-47), Joseph Hadyn

**FIGURE 1:**
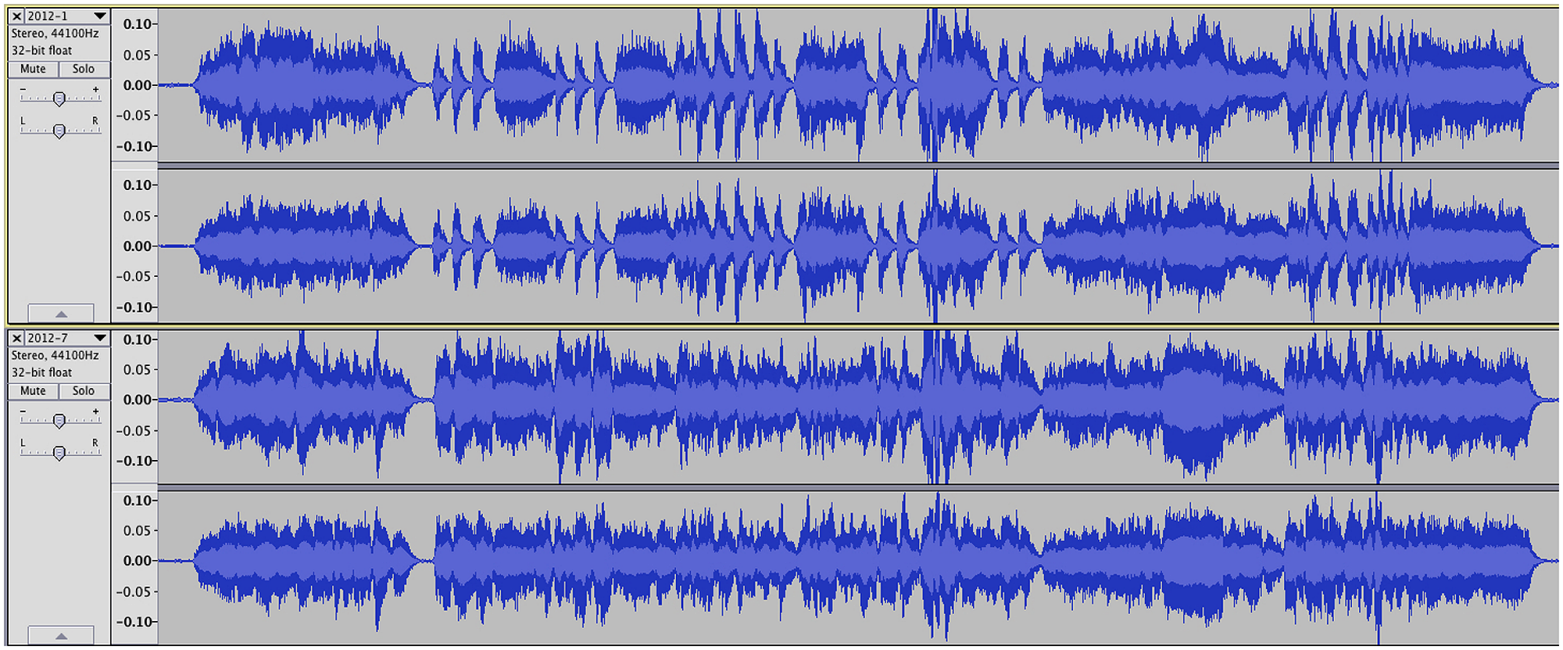
**Sample waveform for high- (upper) and low-contrast (lower) articulation performance**.

One of the researchers arranged, rehearsed, and recorded each of the four excerpts, creating one high (E+) and one low (E-) expression version, yielding a total of eight different performances. For each pair (E+, E-), the conductor utilized a metronome and headset to ensure tempo consistency. During the recording process, performers were instructed to maximize or minimize variance of the target characteristic (articulations or dynamics) and perform all other variables as similarly as possible. In other words, excerpts focusing on articulation were performed at a consistent dynamic level and excerpts focusing on dynamics were performed with consistent articulation throughout. For the low-expression/neutral (E-) condition, we instructed the performers not to vary articulation values, dynamics, or any other expressive elements. Visual waveform analysis performed on each excerpt through the Audacity audio editor (sourceforge.net) showed distinct contrast between performance conditions along the target characteristics (**Figure 1**). Additionally, the researchers (all university music faculty members or graduate music instructors) reviewed the audio segments and agreed that there were clear contrasts between performance conditions and that tempo was consistent across each excerpt pair.

**FIGURE 2:**
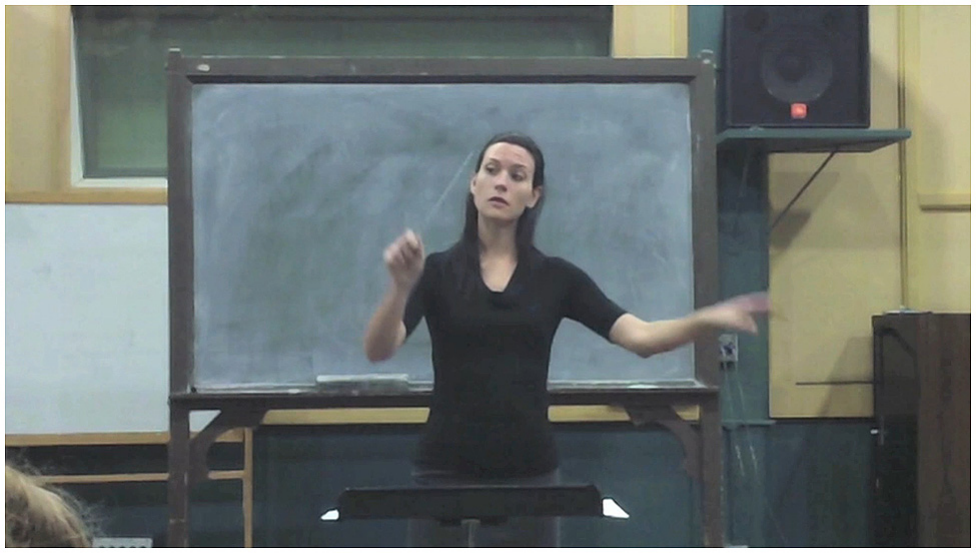
**Screenshot of conductor video**.

For the video portion of the stimuli four graduate conducting students – two male and two female – were recruited to assist in the study. Each conductor was given scores and recordings of the four segments and provided with guidelines of high and low expressivity conducting (Byo and Austin, 1994). Using Flip HD and Zoom H3 camcorders, the conductors were video recorded conducting each excerpt synchronized with a purpose-recruited live ensemble (**Figure 2**). Similar to the original ensemble recordings, conductors were recorded for each of the four excerpts showing a high (C+) or low (C-) level of expressive gesture appropriate for the target characteristic, resulting in a total of eight video segments for each. Ensemble members changed position every two excerpts and conductors changed clothing for each segment to control for any performer-based environmental factors and to create the impression that performances were recorded by different ensembles at different rehearsal sessions. Researchers not familiar with the conductors and blind to the conditions – each an experienced university-level ensemble conductor – reviewed the completed video recordings to ascertain that there were clear contrasts between C+ and C- conducting conditions.

The video segments were imported into iMovie (Apple Inc.) and stripped of ambient audio. We then paired the prerecorded high- and low-expressivity audio segments with the high- and low-expressivity video segments to create fully crossed conductor–ensemble performance combinations of all conditions (C+/E+, C+/E-, C-/E+, and C-/E-) creating a total of 64 combined audio/video segments. To ensure that the test was of a reasonable duration, we selected 32 segments for use representing maximal distinction between high and low level of expressive gesture while maintaining even distribution across conductors and conditions. To prevent identical video stimuli from appearing within a single test administration, the 32 segments were divided into two equivalent 16-item test forms, each including two items from each condition. Presentation order was randomized with the stipulation that neither the same conductor nor the same music excerpt would appear successively. To allow time for participants to respond, the audio/video segments were interspersed with a screen displaying “Please Respond” for 8 s. Using iDVD (Apple Inc.), we burned each order onto a separate DVD.

To ensure that participants were unfamiliar with the conductors and ensemble members in the videos, data collection took place at a different institution in a different region of the United States from where the stimulus materials were prepared. Participants (*N* = 285) were undergraduate students (music majors, *n* = 77; non-majors, *n* = 208) enrolled in music courses at a midsized Southern university. Data collection took place in class settings with groups randomly assigned to one of the two test forms (*n* = 143 and 142). Participants were asked to watch the stimuli and evaluate the group’s performance on several characteristics using a 10-point Likert-type scale (*Poor* to *Excellent*). Qualities for evaluation included articulation, dynamics, and expressivity. To obscure the emphasis on these specific target characteristics, participants also evaluated performance tempo and ensemble technique, qualities bearing minimal relationship with the conductor’s gestures. We did not define any of these qualities for the participants. However, because these constructs were addressed as part of the courses in which participants were enrolled, we felt that the terms would be adequately understood. In the event further clarification was needed, participants were invited to ask questions prior to the beginning of the test. Test administration took approximately 20 min with procedures approved by and carried out in accordance with the university’s Institutional Review Board.

## RESULTS

For each of the four pairings of conductor and ensemble expressivity (C+/E+, C+/E-, C-/E+, and C-/E-), we calculated mean scores for participants’ evaluations of articulation, dynamics, and expressivity. Prior research demonstrated that participants rated ensemble performances as more expressive when the performances were associated with more expressive conducting, even when those performances were identical (Morrison et al., 2009; Price and Mann, 2011; Morrison and Selvey, in press; Price et al., in press). To determine whether the present data were consistent with previous findings, we compared expressivity scores using a repeated-measures analysis of variance with the four conductor/ensemble expressivity pairings as a within-subject variable and major status (music major, non-major) and test form as between-subject variables; alpha level was set at 0.01. There was a significant main effect for expressivity (*F*(3,279) = 144.97, *p* < 0.001) with a strong effect size (partial η^2^ = 0.61; Cohen, 1988). Using Bonferroni correction for multiple comparisons, we found significant differences between each pair of the four conditions except between the C+/E- (high-conductor/low-ensemble) and C-/E+ (low-conductor/high-ensemble) conditions (**Table 2**). Data indicate that participants judged ensemble performances as being more expressive when matched with conducting that featured greater visual contrast along a specific expressive dimension (articulation or dynamics), regardless of whether the ensemble performed with or without commensurate expressive contrast.

**Table 2:**
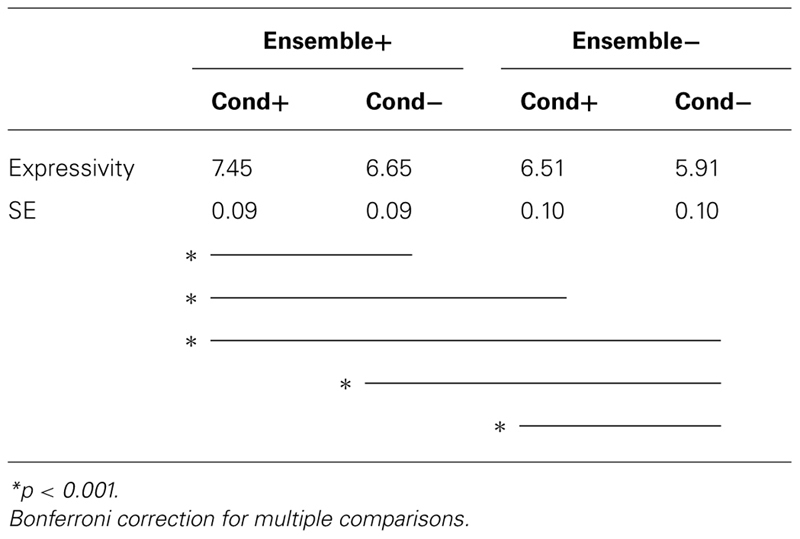
Mean ensemble evaluations by target and condition (underscores show significant pairwise comparisons).

There was a significant interaction between expressivity and major (*F*(3,279) = 5.99, *p* < 0.001). Regardless of the ensemble’s performance, music majors’ responses to low-expressivity conducting were more negative than those of the non-majors. Similarly, a significant interaction between expressivity and test form (*F*(3,279) = 4.56, *p* < 0.001) revealed that responses to low-expressivity conducting on one test form were more negative than for those on the other. The effect sizes of the two interactions were small (partial η^2^ = 0.06 and 0.05, respectively) and neither interaction resulted in any alteration to the relationships among the evaluations. The main effects for major and test form were neither significant nor was the three-way interaction of expressivity by major by test form. The overall lack of difference between responses of music majors and non-majors supported our assumption that participants of varied levels of formal music training would understand and be comfortable with the evaluation task.

**FIGURE 3:**
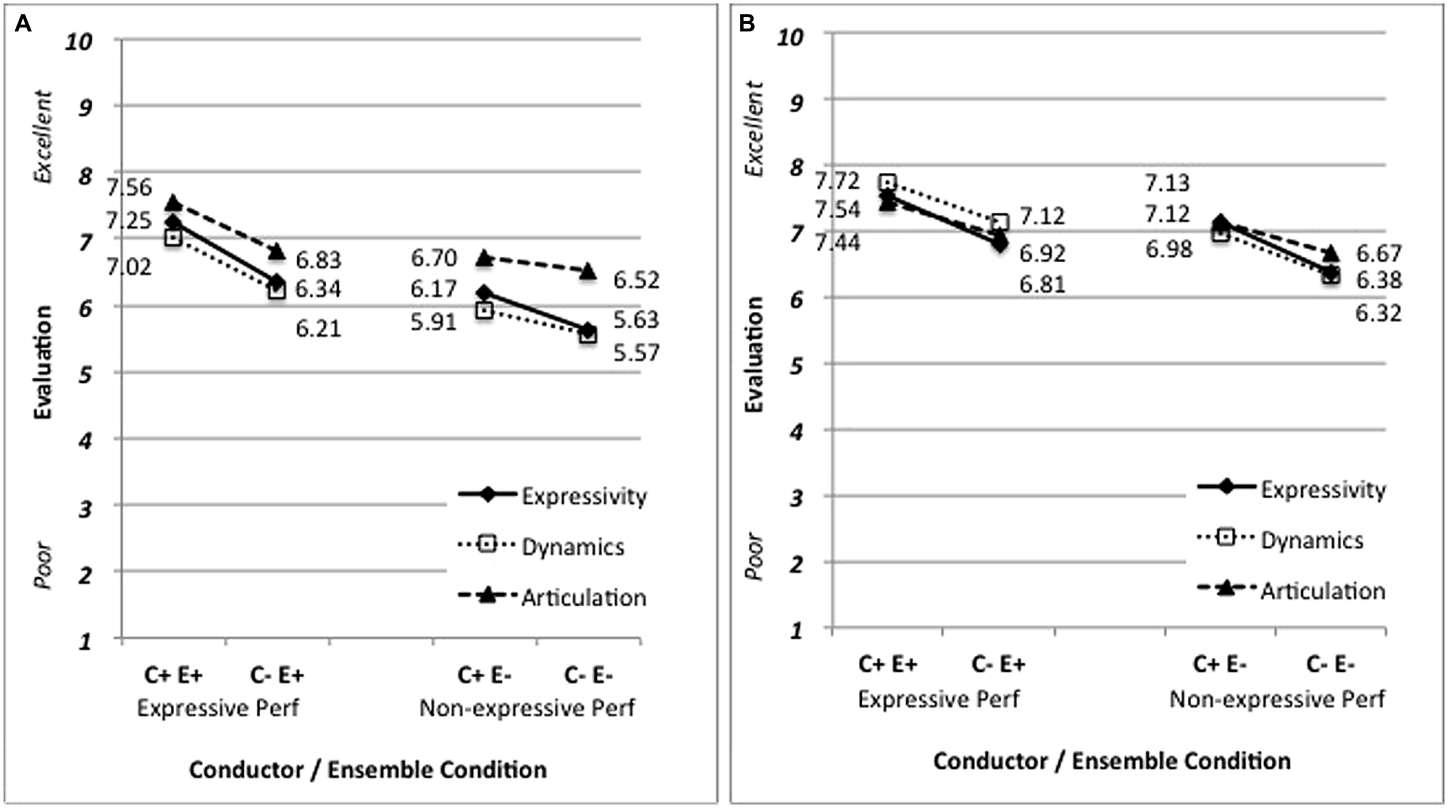
**Mean evaluations for examples targeting (A) articulation and (B) dynamics**.

Having determined that evaluations of ensemble expressivity varied depending on the visual information provided by the conductor, we then examined the relationship between these evaluations and those for the target parameters of dynamics and articulation. We separated the 16 items for each of the two targets and calculated mean responses for each pairing of conductor and ensemble expressivity (C+/E+, C+/E-, C-/E+, and C-/E-) resulting in a set of four articulation and four dynamics scores for each participant. We also calculated a mean for each pairing of expressivity scores corresponding to each characteristic. This resulted in four expressivity scores for the articulation examples and four for the dynamics examples. Articulation scores were significantly and positively correlated with expressivity scores (Pearson’s *r* = 0.72, *p* < 0.001); there was also a significant positive correlation between dynamics and expressivity scores (*r* = 0.85, *p* < 0.001). As with expressivity evaluations, both dynamics and articulation were evaluated more positively for performances featuring more expressive conducting (**Figure 3**).

Interestingly, we also observed comparably strong correlations among examples not highlighting the target variable: articulation and expressivity scores were significantly correlated for dynamics items (*r* = 0.78, *p* < 0.001) and dynamic and expressivity scores were significantly correlated for articulation items (*r* = 0.83, *p* < 0.001; **Figure 3**). Indeed, across all items there was a significant positive correlation between expressivity scores and evaluations of dynamics or articulation (*r* = 0.84 and 0.73, *p* < 0.001, respectively). It appears that participants’ evaluations of any particular performance parameter tended to reflect their overall evaluations of expressivity. To examine this further, we analyzed responses to the two distractor items: performance tempo and ensemble technique. Though these aspects of ensemble performance are arguably unaffected by conductor gesture, participants’ evaluations of these two parameters showed a strong relationship to assessment of expressivity (*r* = 0.72 and 0.85, respectively).

To further clarify the relationship between expressivity and the target characteristics, we used a regression analysis (stepwise) to determine the combined contribution of articulation and dynamics scores, conducting condition, ensemble performance condition, music major status, and test form to expressivity evaluations. The final model (**Table 3**) predicted 75.1% of the total variance (*R^2^* = 0.75, *F*(4,2276) = 1720.38, *p* < 0.001) and included all variables except ensemble performance of dynamics, ensemble performance of articulation, music major status and test form. Articulation and dynamics evaluation scores alone predicted a full 74.8% of the variance with minimal though statistically significant contributions by conductor gesture. All tolerances were well within accepted limits (range 0.45–1.00) indicating that the model was not compromised by multicollinearity.

**Table 3:** Summary of final regression model predicting expressivity evaluations.

Variables	*B*	*SE B*	*b*	*R^2^* change
Dynamics evaluation	0.64	0.02	0.64*	0.712
Articulation evaluation	0.31	0.02	0.27*	0.036
Conductor dynamics	0.44	0.09	0.06*	0.001
Conductor articulation	0.36	0.09	0.04*	0.002

*p < 0.001.

## DISCUSSION

It was the purpose of this study to examine whether differences in evaluations of ensemble expressivity observed for performances featuring highly or minimally expressive conducting would be evident within evaluations of specific aspects of ensemble performance. The present data suggest that, at least in terms of articulation and dynamics, such a relationship between visual and auditory information persists and is strongly and positively correlated with evaluations of overall expressivity. This is consistent with previous data suggesting that greater and more varied movement among performers is associated with more expressive performance (Luck and Toiviainen, 2010). Furthermore, there is evidence that visual information may also affect evaluations of aspects of ensemble performance that are largely unrelated to gestural communication.

In the case of conductors – performers whose movements have no direct effect on the sounds being created – previous research has determined that the degree of gestural expressivity exhibited has a significant impact on the way in which musical performances are evaluated (Morrison et al., 2009; Price and Mann, 2011; Napoles, 2013; Morrison and Selvey, in press; Price et al., in press). In the present study, we also observed that evaluations of overall ensemble expressivity were higher in cases where the conductor exhibited more pronounced variability of gesture. Across identical ensemble performances, significant differences in evaluations were evident between expressively conducted excerpts and those conducted in a more neutral manner. In cases where the ensemble performance itself was more expressive (as defined by greater variability among dynamics and articulation elements), low conducting expressivity resulted in evaluations that were no different than evaluations of low-expressive performances featuring high-expressive conducting.

At least among these particular selections, expressivity of conducting gesture (or lack thereof) either enhanced or detracted from participants’ impressions to such a degree that evaluations of qualitatively different performances could be rendered essentially equal. Morrison and Selvey (in press) found that less-expressive conducting resulted in a significant drop in expressivity ratings of choral performances compared to performances presented in an audio-only format. In contrast, Rodger et al. (2012) observed an increase in evaluations of quality when video of an expert clarinetist was matched with audio of a novice performer but no corresponding decrease in ratings when novice video was matched with expert audio. In terms of direction and magnitude, the manner in which visual information affects music evaluations may be dependent on contextual variables such as performance scale (individual versus ensemble) and general level of musical accomplishment demonstrated by the performers. Moreover, such apparently inconsistent results could indicate that the inclusion of visual information gives rise to a percept unique to themultimodal music interaction rather than simply having additive or decremental effect.

Our results are consistent with Tsay’s (2013) findings in which both novice and expert evaluators were most able to replicate piano competition outcomes when using only visual elements – rather than audio or combined audio and video – of performances by concert pianists. Similarly, raters with varied levels of musicianship accurately evaluated the quality of clarinet performances after seeing the performance without sound (Rodger et al., 2012) though, in this case, differences were more pronounced when audio was included. Although participants in the present study were able to both see and hear the performances, the lack of difference between evaluations of expressive ensemble performances featuring less-expressive conducting and less-expressive performances featuring more-expressive conducting further substantiates the apparently critical role of visual information.

Studies of the relationship between performer movement and viewer/listener perception extend into specific aspects of a musical performance. Evaluation of seemingly unambiguous characteristics as melodic direction, harmonic content ,and note length (Thompson et al., 2005; Schutz and Lipscomb, 2007) vary depending on the accompanying visual information. In the present study, we isolated similarly specific musical parameters germane to instrumental ensemble performances in the Western tradition – articulation and dynamics – and denoted by an agreed upon range of conducting gestures. In contrast to previous research that reported judgments specifically related to the magnitude of the target variable (duration of pitches, for example), we asked participants to judge the general accuracy of various parameters of the ensemble performances. Again, conductor gesture had a significant effect on evaluations of performances, even when participants were asked to specifically evaluate the quality of an ensemble’s performance of articulation and dynamics. Ensemble articulation and dynamics was rated as better when the variability was represented visually as well as aurally. However, even with an absence of variability in the performance, evaluations were higher when conductor variability was provided. Further study is warranted on the degree to which conducting gesture may have an impact on specific discrimination judgments of ensemble performance characteristics.

Participants’ evaluations did not appear to make distinctions between specific aspects of performance. The strong relationship between evaluation of general and particular performance characteristics echoes findings of Price and Mann (2011) who reported a similarly strong relationship between judgments of *expressivity* and performance *quality*. The relationship observed here between evaluations of dynamics and overall expressivity was strong even for examples in which dynamic variability played a minimal role. In fact, this correlation remained stronger than that between expressivity and articulation for examples in which contrasts within the latter parameter figured prominently in the performance. Admittedly, it cannot be assumed that listeners equated how well the ensemble executed a particular facet of a performance with how variable that facet was within the excerpt. All evaluations were, not surprisingly, quite positive given that all examples were performed with a high level of technical accuracy and mature characteristic tone. Furthermore, because participants did not have access to printed scores, it is possible that the absence of dynamic or articulation variability may not have been viewed as a shortcoming in the performance.

It is possible that the participants may have interpreted assessments of articulation and dynamics as having a similar meaning to an assessment of expressivity, particularly those participants possessing less formal music training. We did not specifically define these terms prior to administration of the evaluation task. However, the lack of difference between the responses of music majors and non-majors suggests that level of expertise was not a factor in participants’ evaluations. In light of the strong correlation between evaluations of expressivity and even the seemingly more removed characteristics of tempo and ensemble technique, it may be that each category of evaluation tapped into a more general construct underlying participants’ perceptions of the performances. Previous research examining affective responses to music has indicated similarly correlated response patterns regardless of the terminology used as evaluation prompts (Madsen, 1997; Lychner, 1998). Here a general sense of expressive performance could have led to positive evaluations of multiple specific performance parameters regardless of their salience in a given performance. That this assessment was then significantly impacted by visual information – the gestures of the ensemble conductor, in the present case – suggests a complex relationship among the many facets of music performance.

Specificity of conducting gestures may be more meaningful to performers than non-performing observers who may simply view gestures as globally more or less expressive regardless of referent. Nevertheless, many of the participants in this study were experienced musicians and presumably aware of the intent behind the conductor gestures. The strong relationship between evaluations of articulation, dynamics and expressivity was as evident among music majors as non-majors. Dynamics in particular appears strongly related to increased movement both in terms of perceived (Bhatara et al., 2011) and actual (Thompson and Luck, 2012) performance. Regardless of the performance, dynamics evaluations demonstrated a stronger relationship with overall expressivity than articulation.

It has been suggested that the power of the interaction between auditory and visual information is derived from the latter’s ability to clarify, accentuate or draw attention to some aspect of the former (Vines et al., 2011). The manner in which movement delineates or amplifies critical musical material present in a performance may allow a listener access to, or at least heighten awareness of, particularly salient affective material. While available data are limited, it seems that in the presence of well-performed affective material, the inclusion of appropriately expressive gestures does not necessarily enhance listeners’ evaluations as much as the presence of unexpressive gestures may detract from the overall sense of expressivity (Morrison and Selvey, in press). In the current study, where performances featured both varied and static realizations of specific expressive elements, one may interpret the generally lower evaluations for less expressively conducted expressive performances as consistent with this finding though this question was not specifically tested. In the case of the performances lacking variability in articulation or dynamics, it is not clear whether a similar effect was operating, whether the higher evaluations for the more expressively conducted examples reflected the effect of a visual enhancement on an otherwise unvaried performance, or whether the combination of visual and auditory information evoked a unique percept differing from that resulting from either individual modality. Just when, where and how a conductor’s gesture can enhance, detract from, or otherwise transform a performance remains a critical area for continued study.

## Conflict of Interest Statement

The authors declare that the research was conducted in the absence of any commercial or financial relationships that could be construed as a potential conflict of interest.
